# Chemotherapy in metastatic gastric cancer: population-based perceptions and practice patterns of medical oncologists

**DOI:** 10.1038/sj.bjc.6601802

**Published:** 2004-04-20

**Authors:** L A Wood, A L A Fields

**Affiliations:** 1 Division of Medical Oncology, Queen Elizabeth II Health Sciences Centre, Room 461, Bethune Building, 1278 Tower Road Halifax, Nova Scotia, Canada B3H 2Y9; 2 Cross Cancer Institute, 11560 University Ave. NW, Edmonton, Alberta, Canada T6G 1Z2

**Keywords:** metastatic gastric cancer, practice patterns, chemotherapy

## Abstract

Although randomised trials in metastatic gastric cancer have shown a survival benefit from chemotherapy, a significant proportion of medical oncologists do not believe that it prolongs survival or improves quality of life, including those who routinely treat metastatic gastric cancer. There was wide variation in what was considered to be ‘standard therapy’ and a statistically significant difference between what medical oncologists consider ‘standard therapy’ and what they use in every day practice.

Gastric cancer remains a major health challenge because of its increasing incidence worldwide and its lethality. The outcome for patients with metastatic gastric cancer (MGC) remains poor with median survivals ranging from 5 to 12 months (Karpeh *et al*, 2001). Enthusiasm for systemic chemotherapy has fluctuated as results have emerged from several phase II and III studies with varying and sometimes inconsistent results in terms of response rate and overall survival. Thus, there is a possibility of significant practice variation in the approach to patients with MGC, both in whether to use systemic therapy and in the choice of regimen. To the authors' knowledge, there is no data from any country that documents the practice patterns and practice variation in the treatment of MGC.

The objective of this population-based study was to quantify the current opinions and practice patterns of Canadian medical oncologists with regards to the benefit of systemic therapy and what systemic therapy would be considered standard in MGC.

## MATERIALS AND METHODS

A two-page, 19-item survey was sent to all practicing medical oncologists in Canada. The survey was designed by two medical oncologists who routinely manage MGC; reviewed by two independent oncology specialists for construct validity; and revised accordingly. All responding medical oncologists answered nine questions pertaining to their demographics, whether they routinely manage patients with MGC, their perception of whether chemotherapy prolongs survival or improves quality of life (QOL), and what they considered ‘standard therapy’ in this disease. Medical oncologists who routinely treat patients with MGC answered 10 further questions about chemotherapy regimens commonly used in ‘every day practice’ and their views on future clinical trials in MGC. Since all Canadian cancer patients are seen in a public health care system and the survey was sent to all Canadian medical oncologists, the intent was to obtain population-based results free from referral and selection bias. The mailing list consisted of the names of medical oncologists obtained from the membership lists of four voluntary medical organisations with the expectation of capturing all medical oncologists. Responses were anonymous. The first mailing was sent out on 5 March 2001 and a second mailing was sent out to all nonresponders on 24 May 2001.

Data was analysed using the SAS-8.2 statistical software package (SAS Institute, Inc., Cary, NC, USA). The *χ*^2^ test for significance for categorical data was used. The Fischer exact test was used when the numbers in the cells were less than 5. Statistical significance was set *a priori* at *P*⩽0.05 and all tests were two-tailed.

## RESULTS

Of 425 surveys that were sent, 256 were returned for a 60% response. Of these, 39 were not evaluated for reasons such as retired or a paediatric practice. Thus, 217 respondents formed the study cohort upon which subsequent analysis was based.

Of the 217 respondents, 121 (56%) indicated that they routinely manage patients with MGC. The number of patients they treated varied from 2 to 20 per year with the most frequent response being five (23%) or 10 (18%) patients per year.

### Use of chemotherapy

Of all respondents, 41% of all oncologists thought chemotherapy prolonged survival in MGC and 59% felt that it improved QOL. Medical oncologists who treat MGC were significantly more likely to perceive a survival benefit (53 *vs* 27%, *P*=0.0001) and QOL benefit (72 *vs* 43%, *P*<0.0001), as shown in Table 1

**Table 1:** Perceptions of chemotherapy on survival and quality of life in metastatic gastric cancer

	**Treat MGC (*n*=118) (%)**	**Do not treat MGC (*n*=94) (%)**	***P*-value**	**All respondents (*n*=212)^a^** **(%)**
*Survival*				
Improves	53	27	0.0001	41
Unsure	18	34	0.007	25
				
*Quality of life*				
Improves	72	43	<0.0001	59
Unsure	20	45	0.0001	31

a Missing data in five.

MGC=metastatic gastric cancer.

.

### Type of chemotherapy

Treatment modalities considered ‘standard therapy’ by all medical oncologists are shown in Table 2

**Table 2:** Opinions on ‘standard therapy’ for metastatic gastric cancer (MGC)

	**Treat MGC (*n*=120) (%)**	**Do not treat MGC (*n*=94) (%)**	***P*-value**	**All respondents (*n*=214)^a^ (%)**
ECF^b^	31	19	0.05	26
ELF^c^	31	17	0.01	25
5-FU/cisplatin^d^	13	10	NS	11
BSC^e^	7	15	0.05	10
Other^f^	9	9	NS	9
Unsure	9	31	<0.001	19

a Missing data in three.

b Epirubicin, cisplatin, 5-FU.

c Etoposide, leucovorin, 5-FU.

d Infusional 5-FU, cisplatin.

e Best supportive care.

f Included 5-FU, doxorubicin, mitomycin C (FAM); 5-FU, doxorubicin, methotrexate (FAMTX); 5-FU, leucovorin.

NS=not significant.

. The most common treatments included epirubicin, cisplatin, 5-FU (ECF) and etoposide, leucovorin, 5-FU (ELF). Medical oncologists who treat MGC were significantly more likely to respond ECF (31 *vs* 19%, *P*=0.05) or ELF (31 *vs* 17%, *P*=0.01) than those who do not treat MGC and less likely to respond ‘unsure’ (9 *vs* 31%, *P*<0.001) or ‘best supportive care (BSC)’ (7 *vs* 15%, *P*=0.05), also shown in Table 2.

There was a statistically significant difference between what medical oncologists who routinely treat MGC considered ‘standard therapy’ and what they most commonly use in ‘every day practice’, as shown in Table 3

**Table 3:** ‘Standard therapy’ *vs* therapy used in ‘every day practice’ in MGC by medical oncologists who treat MGC

	**Standard therapy (*n*=120) (%)**	**Therapy in every day practice (*n*=118) (%)**	*P*-value* (%)
ECF	31	27	0.0001
ELF	31	39	<0.0001
5-FU/cisplatin	13	16	
Best supportive care	7	4	<0.0001
Other	9	15	<0.0001
Unsure	9	0	

* *P*-value: the Fischer exact test.

MGC=metastatic gastric cancer.

. Most notably, more ELF and less ECF was routinely used.

### Clinical trial information

Clinical trials in MGC were considered high priority by 81% of medical oncologists who routinely manage MGC with preference given to phase III trial participation. Opinions regarding the reference arm for future phase III trials varied with the most common responses being ECF (34%), ELF (22%), and BSC (17%).

## DISCUSSION

Despite significant research in gastric cancer, the overall prognosis remains poor. Systemic therapy for MGC has evolved over the past 4 decades. Initially single agents such as 5-FU, doxorubicin, mitomycin C and cisplatin were shown to have activity. This led to the development of combination chemotherapy such as 5-FU, doxorubicin and mitomycin C (FAM) and 5-FU, doxorubicin and carmustine (FAB). Comparative studies of these combinations to single agents did not show an improvement in RR or survival (Cullinan *et al* 1985; Levi *et al*, 1986).

Potentially more effective combinations such as 5-FU, doxorubicin and methotrexate (FAMTX), etoposide, doxorubicin, cisplatin (EAP), ELF and ECF were studied with varying response rates (Klein, 1989; Preusser *et al*, 1989; Findlay *et al*, 1994; di Bartolomeo *et al*, 1995). The value of combination chemotherapy was again questioned. In fact, in 1990, the Swedish Consensus Conference stated, ‘The use of chemotherapy has no place in the routine care of patients with advanced gastric cancer’ (Consensus Statement, 1990).

Owing to these emerging views, four randomised trials of combination chemotherapy *vs* BSC were conducted. All showed statistically significant improvement in survival with combination therapy. These trials included a comparison of BSC to FAMTX (median survival 3 *vs* 9 months) (Murad *et al*, 1993); 5FU, epirubicin, methotrexate (FEMTX) (median survival 3.1 *vs* 12.3) (Pyrhonen *et al*, 1995); 5-FU, leucovorin±etoposide (median survival 5 *vs* 8 months) (Glimelius *et al*, 1997); and 5-FU, leucovorin, and epirubicin (median survival 4 *vs* >7.5 months) (Scheithauer *et al*, 1995). An improvement in QOL with chemotherapy using the EORTC QLQ-30 version 1.0 (45% prolonged or high QOL *vs* 20%, *P*<0.01), as well as physician's perception of improved QOL (55 *vs* 20%, *P*=0.03) was shown (Glimelius *et al*, 1997). Although these studies were small, used different chemotherapy, and have some methodological limitations, their results were consistent, with median survivals increasing from 3–5 months with BSC to 8–12 months with combination chemotherapy. Based on these studies, it is reasonable to conclude that combination chemotherapy does offer a survival benefit to patients with MGC.

If there is a benefit from chemotherapy, the obvious question is, which combination is best? Three of the most notable comparative trials show that FAMTX was superior to FAM (median survival 42 *vs* 29 weeks) (Wils *et al*, 1991); ECF was superior to FAMTX (median survival 8.9 *vs* 5.7 months) (Webb *et al*, 1997); and neither FAMTX nor ELF nor infusional 5-FU/cisplatin (FUP) were superior (median survival 6.7 *vs* 7.2 *vs* 7.2 months) (Vanhoefer *et al*, 2000). The QOL was assessed in the ECF *vs* FAMTX trial and found to be equal with the exception of superior global QOL scores with ECF at 24 weeks. Considering these three trials, one might conclude that ECF is superior although it has not been directly compared to ELF or FUP.

Despite randomised trials showing a survival benefit from chemotherapy, this survey showed that less than half of all medical oncologists believe that chemotherapy improves survival and just over half believe that it improves QOL. Interestingly, only 53% of medical oncologists who routinely manage MGC felt that chemotherapy improved survival. This raises the question of why these varying perceptions of benefit exist. This survey did not address the reasons behind these differences. Some reasons may include that medical oncologists do not feel the evidence is strong enough, that the observed benefit does not meet their own predefined threshold of benefit, that the studies were methodologically flawed or that patient numbers were too small. Perhaps the studies referred to are not widely known or that the known information is simply not put into clinical practice.

This survey also identified wide variation in what is considered ‘standard therapy’ for MGC. To help explain the variation seen in this survey, one simply has to look at the literature. Based on the improvement in survival with ECF over FAMTX, the Royal Marsden Hospital concluded ‘ECF should be regarded as the standard treatment in advanced esophagogastric cancer’ (Webb *et al*, 1997). Given the comparable outcomes with ELF, FAMTX and FUP, the EORTC concluded that ‘based on their low activity in advanced gastric cancer, none of these regimens can be regarded as standard treatment’ (Vanhoefer *et al*, 2000). The National Comprehensive Cancer Network has proposed 5-FU-based, cisplatin-based, taxane-based or irinotecan-based combinations as acceptable choices (National Comprehensive Cancer Network, 2003). In an editorial, Ajani points out that ‘5-FU alone or with S-1 is standard therapy in Japan, but 5-FU plus cisplatin is also used frequently. 5-Fluorouracil plus cisplatin is frequently used in Korea, Japan, many South American countries and many European countries. The ECF is considered as the standard in a few European countries and perhaps in Canada’ (Ajani, 2000). Since this survey, two other randomised phase III studies have been published showing no survival benefit of FUP over 5-FU alone (Ohtsu *et al*, 2003) or protracted venous infusion 5-FU, cisplatin, and mitomycin over ECF (Ross *et al*, 2002). Based on these results, the Japanese Clinical Oncology Group is using 5-FU alone as their reference arm and the Royal Marsden Hospital continues to use ECF as their reference arm for future studies.

This survey also showed a difference between what medical oncologists considered ‘standard therapy’ and what they used most commonly in ‘every day practice’. This was an unanticipated result and the survey did not address the reasons for this difference. Perhaps certain chemotherapy regimens are felt to be too toxic or complex or once again, that the known information is simply not put into clinical practice.

Metastatic gastric cancer is a global disease with a poor prognosis. New therapeutic regimens must reproducibly produce median survivals of greater than 12 months to be convincingly superior to the older regimens and data must come from well-designed studies. Alternatively, given that there is no consensus on the reference arm for future phase III trials some would argue that comparative studies be put on hold with efforts instead concentrated on new drug development and novel approaches. The findings of this study suggest that once significant breakthroughs are made, the dissemination of such information is of importance and should not be overlooked.
